# Emotional Memory and Amygdala Activation

**DOI:** 10.3389/fnbeh.2022.896285

**Published:** 2022-06-13

**Authors:** Margaret M. Bradley, Nicola Sambuco

**Affiliations:** Center for the Study of Emotion and Attention, University of Florida, Gainesville, FL, United States

**Keywords:** emotion, amygdala, hippocampus, episodic memory, perception, imagery, repetition suppression, repetition enhancement

## Introduction

Determining the neural correlates of emotional memory is critical for understanding both healthy and psychopathological emotional processing, and meta-analyses summarizing dozens of individual studies (e.g., Murty et al., 2010; Dahlgren et al., 2020) report enhanced amygdala activity during both encoding and retrieval of emotional, compared to neutral, stimuli, implicating the amygdala as central in enhanced memory for emotional items (see also Phelps and Anderson, 1997). In this Opinion article, we raise a number of issues regarding amygdala activation and emotional episodic memory. First, the majority of fMRI studies investigating emotional episodic memory assess memory for emotional scenes or facial expressions (e.g., Dahlgren et al., 2020), but then generalize findings to memory for the wide variety of emotional events encountered in the natural world. In the brain (and body), however, differential activity due to emotional processing can vary as a function of the specific emotional challenge (Bradley, 2000; Sabatinelli et al., 2011; Bradley and Lang, 2018; Sambuco et al., 2020a), raising questions regarding the generality of amygdala activation in emotional processing. Second, effects of emotion at retrieval are assessed using a number of different fMRI contrasts, with some potentially including differences in functional activity that are related to emotionality, but not necessarily to memory, whereas, for others, excellent memory performance precludes accurate assessment of unique effects of emotion. Below, we more fully consider these issues, and discuss implications for both theory and data.

### Emotional Challenge

**Figure 1:**
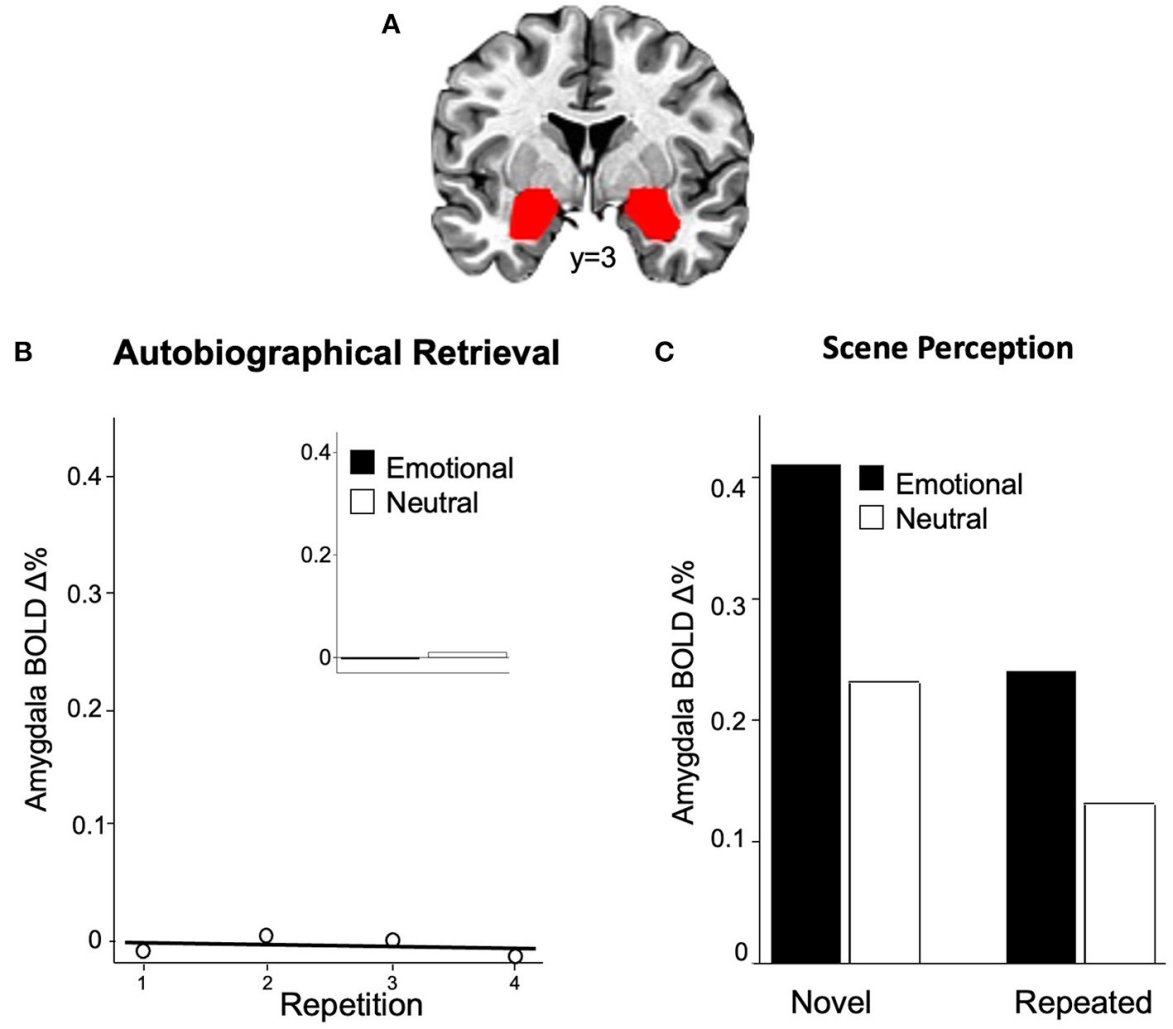
Enhanced functional brain activity in the amygdala **(A)** is not found when repeatedly retrieving emotional autobiographical events **(B)**, with no difference between emotional and neutral content [**(B)**, inset]. Amygdala activation is consistently found when [**(C)**, left] encoding emotional, compared to neutral, pictures but is significantly reduced (repetition suppression) when the same scenes are repeatedly retrieved [**(C)**, right].

The extent to which amygdala activation is involved in emotional episodic memory, whether during encoding or retrieval, relies, first of all, on its reliable activation across emotional contexts. Whereas enhanced amygdala activity is a key finding when viewing emotional, compared to neutral, scenes or faces (see Sabatinelli et al., 2011 for a meta-analysis), significant amygdala activation is not reliably obtained when retrieving personal emotional memories, which is central in both healthy and psychopathological functioning. Thus, whereas some studies report amygdala activation during emotional autobiographical retrieval (e.g., Britton et al., 2005; Sharot et al., 2007), others do not (Nadel et al., 2007; Piefke et al., 2008; Svoboda and Levine, 2009; Lanius et al., 2010). To re-address this issue, we asked participants to retrieve and imagine the same pleasant and unpleasant autobiographical events repeatedly (four times) across a scanning session while amygdala activation (Figure 1A) was measured (Bradley et al., 2022). Figure 1B illustrates the pattern of blood-oxygen-level-dependent (BOLD) change in the amygdala across repeated retrieval of emotional autobiographical events, which did not prompt any significant amygdala activation, regardless of repetition.

It is possible that operations of spatial smoothing and/or standardization in previous studies misattributed activation of the anterior hippocampus, which lies just adjacent to the amygdala and is reliably enhanced during autobiographical retrieval (Sambuco et al., 2020a; Bradley et al., 2022), to the amygdala. To test this, we assessed amygdala activation as it varied with emotional challenge in a repeated-measures design in which the same participants viewed emotional (or neutral) scenes and retrieved emotional (or neutral) events (Sambuco et al., 2020a). Identical pre-processing steps of spatial smoothing and standardization were conducted for both sets of functional data, allowing a direct assessment of the extent to which different emotional challenges prompt enhanced activation in the same or different neural regions. Replicating many previous studies, significant enhanced BOLD activity was found in the amygdala when viewing emotional, compared to neutral, scenes, as well as in the inferior frontal gyrus and the visual cortex (for a meta-analysis, see Sabatinelli et al., 2011). On the other hand, ROI analyses confirmed that retrieving personal emotional, compared to neutral, events did not differentially or significantly activate the same amygdala region, but that activation of the adjacent anterior hippocampus was enhanced during emotional autobiographical retrieval (Sambuco et al., 2020a; see also Sambuco et al., 2022).

Differences in emotional reactivity due to the specific emotional challenge are also routinely found in psychophysiological reactions, with, for instance, cardiac deceleration (slowing), a parasympathetically mediated response that facilitates sensory perception, found during aversive visual perception, and cardiac acceleration (speeding), a sympathetically mediated response that supports action preparation, found during aversive mental imagery (Lang, 1979; Vrana et al., 1986; Bradley and Lang, 2018). Thus, finding diverse patterns of neural activity when encoding and/or retrieving different emotional challenges is not surprising, and both human and animal data document a variety of brain regions activated during emotional processing, including the insula, basal ganglia, striatum, cingulate, cerebellum, and more (Gasquoine, 2014; Wang et al., 2016; Adamaszek et al., 2017; Pierce and Péron, 2020). And, whereas early studies reported significant amygdala involvement when retrieving cues associated with the presentation (Phelps et al., 2004) or prediction of electric shock (Phelps et al., 2001; Alvarez et al., 2011), more recent investigations do not find significant amygdala activation in either fear conditioning (Fullana et al., 2016; Visser et al., 2021) or when under threat of shock (Kirlic et al., 2019; Sambuco et al., 2020b,c).

### Retrieval Contrast

For emotional challenges (such as scene perception) that include significant amygdala activation, studies assessing retrieval-related functional activity have utilized a number of different contrasts to support the proposed relationship between emotionality and amygdala enhancement. In a recent meta-analysis for example (Dahlgren et al., 2020), the majority of included retrieval studies directly compare functional brain activity at recognition for correctly recognized emotional and neutral items (“hits”), assuming that differences reflect enhanced memory for emotional stimuli. However, functional contrasts that compare emotional and neutral hits can include differential amygdala activation due to differences in emotion, rather than related to retrieval. That is, similar to encoding, these retrieval cues, which are perceptually processed prior to contacting an episodic memory representation, could prompt differential amygdala activation that is unrelated to episodic retrieval.

A second common comparison computes “difference in memory” (DM) maps for emotional (or neutral) items that contrasts functional activation between correctly remembered (“hits”) and forgotten items (“misses”). For emotional scenes, however, immediate recognition is almost perfect (~90% accuracy; Ferrari et al., 2013; Weymar et al., 2018), leaving very few trials available for constructing maps of emotional “misses,” which greatly reduces the reliability of both the statistical contrast and resulting conclusions (Chen et al., 2022). In fact, even when immediate recognition was assessed for hundreds of emotional and neutral scenes, performance was so high that Kalpouzos et al. (2012) were not able to construct DM maps for emotional “misses.” Moreover, in a final step, studies using DM contrasts compare emotional and neutral DM maps, but, as when comparing emotional and neutral hits, differences due to emotionality, rather than memory-related processes, may remain in these functional contrasts.

An alternative way to assess the effects of emotion at retrieval, while controlling for differences in sheer emotionality, is to repeat the same emotional items. In particular, when repetitions are distributed across an imaging session, both theory and data support the hypothesis that these spaced repetitions engage spontaneous episodic retrieval (e.g., Greene, 1989; Hintzman, 2010), in which the re-presentation of a cue, following perceptual processing, activates its prior episodic occurrence. Moreover, because immediate recognition for pictures is almost perfect with just a single prior presentation, repetitive presentation will prompt successful recognition, even in the absence of an explicit performance measure. If amygdala activity is specifically enhanced during episodic retrieval of emotional cues, increased amygdala activation should be found for repeated scenes, compared to when the same emotional cues are novel.

To assess this hypothesis, Bradley et al. (2015) presented novel and repeated emotional and neutral scenes distributed across a session (4x), finding the expected enhanced amygdala activation during initial encoding (novel) of emotional, compared to neutral, scenes (see Figure 1C, left). Moreover, amygdala activation continued to be greater when viewing emotional, compared to neutral, scenes, for repeated stimuli. Importantly, however, as illustrated in Figure 1C (right), compared to encoding, repeated presentation of the same emotional scenes elicited significant repetition suppression, in which amygdala activity was reduced, rather than enhanced, at retrieval. Similar repetition suppression in the amygdala is found during both explicit and implicit scene recognition (Weymar et al., 2018) and following the repetition of emotional and neutral faces (Ishai et al., 2004). In general, repetition suppression effects during episodic retrieval have been variously interpreted as indexing neural priming, perceptual sharpening or information accumulation (e.g., Schott et al., 2005; Yassa and Stark, 2008; Rugg and Vilberg, 2013), raising questions regarding its specific role during episodic retrieval. Taken together, however, although amygdala activation is greater when retrieving emotional, compared to neutral, scenes, this may reflect differences in emotionality, rather than memory, and does not show the expected enhancement, but rather suppression, when compared to encoding.

## Discussion

As frequently noted, memory performance is generally enhanced for emotional, compared to neutral, information (Bradley et al., 1992; Hamann et al., 1999; Dolcos et al., 2005, 2017; Kensinger and Schacter, 2008), and amygdala activation at encoding and/or retrieval is often proposed as a critical mechanism. Much of the supporting data, however, arise from studies assessing memory for visual scenes or faces, whereas amygdala activation is not a general finding across emotional challenges. Thus, emotional memory accounts that include a key role of the amygdala, based primarily on data from emotional challenges that include significant activation of this region (e.g., amygdala-frontal regulatory circuit, Hartley and Phelps, 2010; Motzkin et al., 2015; amygdala-sensory connections, Mather and Sutherland, 2011; Bowen et al., 2018) will not necessarily generalize to memory in other emotional contexts. Elucidating the neural mechanisms important in emotional episodic memory will instead first need to carefully consider the nature of the emotional challenge, as is generally the case in the study of emotion (Bradley, 2000; Bradley and Lang, 2018), with broader generalities proposed when the data confirm cross-context commonalities.

In addition, regardless of the specific emotion challenge, the nature of the functional contrast used to assess emotional differences at retrieval is also critical, as excellent memory performance for emotional stimuli may rule out contrasts requiring a reasonable number of misses (such as DM contrast). More importantly, comparing functional maps at retrieval for emotional and neutral items is problematic, as these can reflect functional activity related to differences in stimulus emotionality that are not associated with episodic memory or retrieval success. Thus, although amygdala activity is higher at retrieval for emotional, compared to neutral, scenes, repetition suppression, rather than enhancement, is found when compared to initial encoding (Ishai et al., 2004; Bradley et al., 2015; Weymar et al., 2018), which doesn't support a prediction of enhanced amygdala activation related to better episodic memory.

Isolating functional activity specific to emotional retrieval is probably better supported by data indicating that successful emotional memory prompts (1) enhanced functional activation at retrieval, compared to encoding, and/or (2) enhanced functional activation that is only apparent at retrieval. Functional enhancement when retrieving emotional scenes and autobiographical memories is reliably reported in large regions of the posteromedial cortex, including the posterior cingulate cortex and precuneus (Kim, 2010, 2017; Bradley et al., 2022), supporting a central and context-independent role in episodic retrieval (e.g., Wheeler and Buckner, 2004; Wagner et al., 2005; Rugg and Vilberg, 2013). During immediate scene recognition (explicit or implicit; Weymar et al., 2018) or following mere scene repetition (Bradley et al., 2015), however, emotional content does not modulate posteromedial activation (perhaps reflecting excellent immediate memory performance for all scenes), but differential effects have been reported in delayed recognition (Ventura-Bort et al., 2020) as well as during autobiographical retrieval (Sambuco et al., 2022). Future studies assessing similarities and differences in neural activation in different emotional challenges, using appropriate functional contrasts, promise to more fully elucidate the neural mechanisms underlying emotional episodic memory.

## Author Contributions

MB and NS conceptualized and wrote initial draft. Both authors contributed to the article and approved the submitted version.

## Funding

This research was supported by NIMH grants MH094386 and MH098078.

## Conflict of Interest

The authors declare that the research was conducted in the absence of any commercial or financial relationships that could be construed as a potential conflict of interest.

## Publisher's Note

All claims expressed in this article are solely those of the authors and do not necessarily represent those of their affiliated organizations, or those of the publisher, the editors and the reviewers. Any product that may be evaluated in this article, or claim that may be made by its manufacturer, is not guaranteed or endorsed by the publisher.
